# The Influence of Self-Efficacy and Locus of Control on Body Image: A Path-Analysis in Aspiring Fashion Models, Athletes and Students

**DOI:** 10.3390/ijerph18116128

**Published:** 2021-06-06

**Authors:** Donatella Di Corrado, Marinella Coco, Maria Guarnera, Nelson Mauro Maldonato, Alessandro Quartiroli, Paola Magnano

**Affiliations:** 1 Department of Sport Sciences, Kore University, Cittadella Universitaria, 94100 Enna, Italy; 2 Department of Biomedical and Biotechnological Sciences, University of Catania, 95123 Catania, Italy; marinella.coco@unict.it; 3 Faculty of Human and Social Sciences, Kore University, 94100 Enna, Italy; maria.guarnera@unikore.it (M.G.); paola.magnano@unikore.it (P.M.); 4 Department of Neuroscience and Reproductive and Odontostomatological Sciences, University of Naples Federico II, 80138 Naples, Italy; nelsonmauro.maldonato@unina.it; 5 Department of Psychology, University of Wisconsin-La Crosse, La Crosse, WI 54601, USA; aquartiroli@uwlax.edu; 6 School of Sport, Health and Exercise Science, University of Portsmouth, Portsmouth PO1 2UP, UK

**Keywords:** body image, eating disorders, stressors, athletes, fashion models

## Abstract

It is widely documented that negative body image is a significant public health concern due to its association with symptoms of disordered eating and worse psychological well-being. The purpose of the study was to develop a path model of intrapersonal dimensions (self-efficacy and internal locus of control) as antecedents of perceived stress toward females’ body dissatisfaction and eating attitude disorders. A total of 300 females, including 100 aspiring fashion models, 100 athletes and 100 students (controls), between 15 and 24 years of age (M = 19.6, SD = 1.85) participated in the study. Measures included level of psychological stress, self-efficacy and locus of control dimensions, body dissatisfaction and eating attitude disorder indices. A path analysis confirmed our research hypothesis. Comparing the three subsamples, we found better fit indexes in the two subgroups with elevated investment on their body image with respect the control group. More specifically, the model in the group of aspiring fashion models showed the best fit index. These results indicated that aspiring fashion models have a strong desire to maintain their low body mass or to become thinner. For this reason, a suitable involvement of expert health workers in the nutritional and psychological field could be extremely essential in the fashion world to maintain a healthier well-being.

## 1. Introduction

Body (image) dissatisfaction can be defined as the depreciation of body weight and physical appearance resulting from a perceived discrepancy between the actual body image and the ideal body image [1]. Several studies indicated that body dissatisfaction is associated with social anxiety, body shame, depression and a wide variety of other experiences that diminish self-efficacy and reduce the quality of life [2,3,4,5]. Valutis and colleagues [6] have found that discrepancies between realistic and ideal body size may influence efforts for modifying, as well as approaches about, body size. Precisely, they suggested that self-efficacy might affect the personal reaction to this discrepancy. In line with efficacy research [7], individuals with large differences between current body size and realistic body size may have ceased making efforts into the issue, implying low self-efficacy related to a person’s belief that they cannot achieve a particular task.

According to Bandura’s social cognitive theory [8], self-efficacy plays an important role in influencing one’s behavior. For example, an individual who believes in being able to produce a desired effect (“I can do it”) controlling their own personal action will be more active and self-determined and more able to deal with certain life stressors. Schwarzer [9] found that a strong sense of personal efficacy is related to better health, higher achievement and more social integration, while a low sense of self-efficacy is associated with depression, stress and reduced motivation and cognitive abilities. There are many elements that influence human behavior (e.g., cognitive, behavioral, personal and environmental), and human functioning is the result of the association among these aspects [10].

Locus of Control (LOC) is an important construct that has a significant impact on Bandura’s self-efficacy theories [11] and how people’s expectations shape their own goals. It indicates the degree to which individuals attribute the cause of the behavior to environmental factors or to their own choices. Individuals with a higher internal LOC perceive control over their circumstances and engage more than do individuals with external LOC, because they believe that their success depends on their will. Furthermore, they tend to feel happier and less stressed [12]. On the contrary, people with a lower internal LOC tend to feel more stressful and perceive themselves to have very little control over their lives, because they attribute successes and failures to external factors, such as luck, destiny, fate or coincidence [13].

The relationship between self-efficacy and locus of control is widely investigated [14,15,16], confirming that they both are interrelated and affect the ability of the person to adjust in a given situation. Some research [17,18,19,20] has shown a positive relationship between self-efficacy and internal locus of control, describing how people who believe they have control over future events are more likely to use that control to achieve a positive result and experience enhanced wellbeing [21]. Thus, internal locus of control and self-efficacy are the dimensions that help a person to meet the requirement of the situation that an individual is dealing with. The significant role that stress plays within psychological theories, such as Rotter’s social learning theory [13] and Bandura’s [8] self-efficacy theory, has been widely explored in the current literature [22,23,24]. For example, individuals with a low internal locus of control and low self-efficacy report experiencing higher levels of psychological and physical complications and worse levels of psychological wellbeing (e.g., depression, increased general stress, anxiety). These individuals perceive themselves as having less control, being more susceptible to external influences and being more likely to focus on obstacles rather than opportunities. However, stress is part of our everyday lives; it is a physical, mental or emotional request that tends to negatively influence individuals’ well-being or functioning, since individuals perceive the situation as stressful, and their resources are inadequate to handle environmental stimuli [25].

Generally, stress can be a primary or secondary contributor to ill health via excessive and sustained sympathetic arousal or through associated behaviors, such as smoking, substance abuse and over- or inappropriate eating. Several studies suggested that stressful life events precede irregular eating and weight control behavior in young females, showing a close association between feelings of stress and body image problems [26,27,28,29]. Cash [30] identified 19% of young females suffer significant distress associated with body dissatisfaction. Researchers found that body (image) dissatisfaction is often recognized as a salient predictor eating disorder symptomatology among women [31,32,33].

The medical term eating disorder describes an illness that is characterized by irregular eating behaviors and severe distress or concern about body weight or shape [34]. Nevertheless, it is important to specify that the probable adverse effects of body dissatisfaction are not limited to the relatively small group of women with clinically diagnosed eating disorder. Instead, the impact of body dissatisfaction is much broader.

In the field of eating disorders, high-risk groups include people born after perinatal complications, individuals who suffered severe negative life events or people whose behavioral patterns will bring to the probable development of an eating disorder, such as excessive dieting, professional pressure to be thin and excessive exercise [27]. According to these last considerations, professional fashion models, dancers, athletes and young females were separately examined.

In the sport field, the majority of studies [35,36,37] reported higher body dissatisfaction in aesthetic sports (synchronized swimming, gymnastics and dance sport), confirming that female athletes experience the sport specific pressure to change their weight, body and appearance. Other research recognized that a large portion of college female student-athletes reported significant body dissatisfaction [38,39,40]. Further studies [41,42,43] showed that professional fashion models bear a higher risk of eating disorders than their peers, because of the constant pressure to maintain a thin shape.

**Figure 1:**
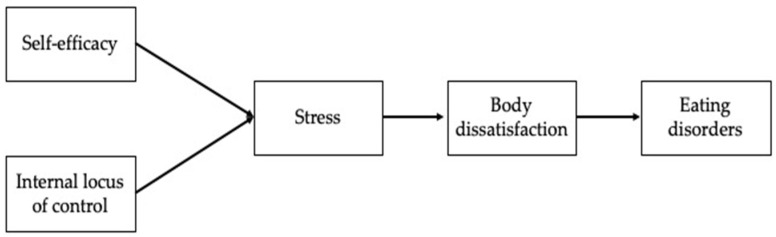
The Hypothesized Model.

To our knowledge, the present study is the first to compare aspiring fashion models to a group of female athletes and a well-matched control group composed of females of the same age and cultural background. Based on these arguments, and given the limited research, the purpose of the study was to develop a path model of self-efficacy and internal locus of control variables as antecedents of perceived stress toward body dissatisfaction and eating attitude disorders. A path analysis was used to examine an integrative model involving intrapersonal dimensions (self-efficacy and locus of control) as predictors of females’ body dissatisfaction and eating disorder through the influence of the perceived stress. The hypothesized model is illustrated in Figure 1. Then, we hypothesized to find differences in the comparison of three subsamples: aspiring fashion models, athletes and controls: more specifically, we expected better fit indexes in the two subgroups with elevated investment on their body image with respect the control group.

## 2. Materials and Methods

### 2.1. Participants

In order to have a sample of participants as homogeneous as possible, participants were included if (a) they were aged between 15 and 24 years; (b) currently, they had to attend school; and (c) they could provide informed consent. Participants were excluded if did not meet aforementioned inclusion criteria. In this study, a total of 300 participants met all inclusion criteria: 100 aspiring fashion models, 100 athletes of different athletic disciplines and 100 female students (controls) between 15 and 24 years of age (M_age_ = 19.6, SD = 1.85). The analysis of variance (ANOVA) did not reveal any significant differences among the three groups in terms of age (F(2, 297) = 1.24; *p* = 0.29; partial *η*^2^ = 0.08.

Aspiring fashion models were young females taking part to the final of the “Look of the Year” (an international competition which annually elects the Model of the Year). Athletes were regional competitors of Track and Field (running, jumping, throwing and sprint). A control group was established in order to compare the study variables among young females who did not have a strong pressure to maintain a thin shape. Thus, students from two high schools and two college of the same age group and cultural background were included in this study. Researchers provided participants, parents/guardians and agents of aspiring fashion models with a full explanation of the goals and the protocol of the study.

Each participant >18 signed a free written consent to participate. A consent form was signed by parents for the younger students and by agents for the aspiring fashion models. All of the procedures were conducted in accordance with the ethics stated in the Declaration of Helsinki, while the University Enna Kore Internal Review Board for psychological research (UKE-IRBPSY-05.21.01) gave ethical permission.

### 2.2. Procedures

First, the responsible researcher contacted the director of the “Look of the Year”. The procedures and objectives of the study were explicated in detail, and authorization for participation was obtained. Afterward, high-school and college principals and head coaches from the athletic clubs were contacted requesting permission to survey student athletes. When consent was received, researchers determined a day and time for them to complete the surveys.

Measurements were executed in group format, with 15–20 participants per session (approximately 35 min.) in a quiet room under the supervision of two researchers. Upon completion of all tests, the anthropometric measurements (in light clothing and without shoes) were taken by a research assistant in another private room. Body weight and height were collected through portable digital scale and stadiometer. All participants performed a battery of tests, and confidentiality of their answers was assured. Aspiring fashion models were tested in a quiet room in the place of competition. The control group was tested in a room in either the university or the school at the end of lecture. Athletes were tested in a private location in the training facilities. During the assessment, no talking was permitted, and no feedback was provided.

### 2.3. Measures

#### 2.3.1. Body Dissatisfaction

Body Dissatisfaction subscale of the Eating Disorder Inventory (EDI–BD; Italian version) [44] was administered. Ten self-report questions are rated on a 6-point scale (from 1 = never to 6 = always). It reflects the belief that specific regions of the body associated with shape change or increased “fatness” at puberty are too large (e.g., hips, thighs, buttocks). A final high average of the scores indicates high body dissatisfaction. The self-report measure has adequate validity when administered in non-clinical adolescent populations. Item reliability was excellent (mean estimates for internal consistency was 0.89). The internal consistency measured for our data was satisfactory, showing an α value > 0.80.

#### 2.3.2. Bulimic Investigatory Test, Edinburgh (BITE)

It is a measure [45] of bulimic pathology consisting of 33 items related to the presence/absence of behaviors and attitude associated with bulimic disorders. We used the validated Italian version of this scale. The items contain: (1) a symptom subscale that assesses the degree of symptoms present and (2) a severity subscale that indicates the severity of binge eating based on frequency. Items are answered in a binary yes–no format. Cronbach’s α in the general population samples was 0.81 in females. The Italian version of BITE is psychometrically sound, and it can be a useful screening tool. Internal consistency of our study was 0.79.

#### 2.3.3. Measuring Psychological Stress (PSM-9)

It is a 9-item, self-administrated questionnaire [46] measuring directly the state of feeling stressed. We used the Italian version of the scale. The internal consistency was satisfactory, showing a Cronbach α coefficient of 0.92 and 0.93. The tool is valid and reliable, and it is suited for assessing stress clinically in the general population and serving as an outcome measure. The internal consistency measured for our data was good, showing an α value > 0.89).

#### 2.3.4. Eating Attitudes Test (EAT-26)

It is a self-report measure [47] of disordered attitudes and behaviors toward eating and body weight control. It is an instrument answered on a 6-point Likert-type scale (1 = never, 6 = always) based on how often the individual engages in specific behaviors. The instrument is scored by assigning points to each response and summing scores for all 26 items. We used the 26-item Italian version of the EAT [48], which proved able to discriminate between clinically diagnosed cases of eating disorders and healthy controls around the original cut-off. A cut-off ≥20 indicates the presence of clinically significant eating pathology. The EAT-26 has been extensively validated across other clinical and non-clinical subgroups from various cultural backgrounds. Mean estimates of internal consistency was 0.86. The value alpha of the internal consistency of our data was 0.73.

#### 2.3.5. The General Self-Efficacy Scale

It is a 10-item psychometric scale [49] aimed to assess optimistic self-beliefs to cope with a variety of difficult demands in life. It states to personal agency, i.e., the credence that one’s actions are responsible for successful outcomes. Typical items are: “Thanks to my resourcefulness, I know how to handle unforeseen situations”, and, “When I am confronted with a problem, I can usually find several solutions.” We used the Italian adaptation of the General Self-Efficacy Scale. The internal consistency was found between alpha = 0.75 and 0.91. The scale is a reliable and valid unidimensional measure across different cultural contexts. In this study, Cronbach’s alpha reliability for the General Self-Efficacy scale was 0.84.

#### 2.3.6. The Mini Locus of Control Scale (MLCS)

It consists of 6 items [50]. People were asked to state their level of agreement according to a 4-point scale: totally (4); enough (3); little (2); not at all (1). It comprises three main subscales: Fatalism, the random play of external circumstances (“There are those who are born lucky and those who are not”; “Without the right opportunities, it is difficult to succeed in life”); Hetero-dependence, the influence exerted by the social environment (“My life is controlled mainly by the influence of other people”; “It is others who decide whether you succeed in your life or not”); and Internality, the personal wills capabilities (“People could do so much more, if only they really tried”; “It’s entirely up to me if I can take advantage of the opportunities life gives me”). The internal consistency of the item scores was satisfactory, showing an α value > 0.81. Its simplicity and validity across a number of studies point to its utilization in this study. The value alpha of the internal consistency of our data was >0.79.

#### 2.3.7. Body Mass Index

Body Mass Index (BMI) was computed from values of weight and height using the standard formula of weight (kg)/height^2^ (m).

### 2.4. Statistical Analysis

The Kolmogorov–Smirnov test showed a normal distribution of the data. Data are expressed as means ± standard deviations(*s*) and the range. A one-way analysis of variance (ANOVA) was performed on the scores of dependent variables (body dissatisfaction, bulimic index, perceived stress; eating attitudes disorder, self-efficacy and locus of control dimensions), comparing the means of each group. A Tukey’s post hoc test was used to describe the existent differences. Moreover, Pearson’s r coefficient was used to determine the relationship between the selected variables.

A path analysis was carried out to test the hypothesized model using Lisrel 8.80 [51]. The goodness-of-fit indices provided by Lisrel include chi-square (χ^2^), comparative fit index (CFI) and the root mean square error of approximation (RMSEA) and the standardized root mean square residual (SRMR). A significant χ^2^ value leads to the rejection of the null hypothesis that the model fits in the population. The CFI provides an evaluation of the difference between an independent model and the specified model. For the CFI, values over 0.90 propose acceptable fit, while values over 0.95 indicate a good fit [52]. According to Browne and Cudeck [53], a RMSEA < 0.09 is still an indicator of a reasonable error of approximation in smaller samples. In the present study, we compared the same model in three subsamples—athletes, aspiring fashion models and controls—using the Akaike Information Criterion (AIC) [54], in which a lower value indicated a superior model fit, compared to models with higher values. Statistical evaluation was carried out using SPSS 23.0 (SPSS Inc., Chicago, IL, USA).

## 3. Results

### 3.1. Descriptive Data, ANOVA Results and Correlations

All 300 participants completed the surveys. Table 1 shows the anthropometric characteristics of the participants. According to WHO Regional Office for Europe, a BMI of 18.5–24.9 is considered normal or healthy for most women; a BMI less than 18.5 indicates underweight.

The one-way analysis of variance (ANOVA) results (body dissatisfaction, bulimic index, perceived stress; eating attitudes disorder, self-efficacy and locus of control dimensions by groups) were significant (Table 2). Specifically for aspiring fashion models, a Tukey’s post hoc test showed significantly higher scores on body dissatisfaction (models vs. controls *p* = 0.001; models vs. athletes *p* = 0.001), perceived stress (models vs. controls *p* = 0.001; models vs. athletes *p* = 0.001), eating attitude disorder (models vs. controls *p* = 0.001; models vs. athletes *p* = 0.001), fatalism (models vs. controls *p* = 0.01; models vs. athletes *p* = 0.001) and hetero-dependence (models vs. athletes *p* = 0.001), while scores on self-efficacy (models vs. controls *p* = 0.001; models vs. athletes *p* = 0.001) and on internality (models vs. controls *p* = 0.001; models vs. athletes *p* = 0.001) came out lower. Furthermore, post hoc comparisons showed significantly lower scores in the group of controls on body dissatisfaction (controls vs. athletes *p* = 0.001) and eating attitude disorder (controls vs. athletes *p* = 0.001), while scores on fatalism (controls vs. athletes *p* = 0.001) and hetero-dependence (controls vs. athletes *p* = 0.001) came out higher. Additionally, self-efficacy (athletes vs. controls *p* = 0.001), and internality (athletes vs. controls *p* = 0.001) were higher in the athletes, while perceived stress (athletes vs. controls *p* = 0.001; athletes vs. models *p* = 0.001) came out lower.

Skewness and kurtosis of the distribution of the total scores of the dimensions of the study were calculated: skewness values were between −0.55 and 1.14; kurtosis values were between −0.33 and 1.10.

The relationships between the variables of the study were analyzed using Pearson’s r coefficient (Table 3).

As can be observed, body dissatisfaction was significantly and positively related to eating attitude disorder and perceived stress and negatively associated with self-efficacy. We found very strong relationships between the intrapersonal dimensions of self-efficacy and internal locus of control, which we assumed as the antecedents in our research hypotheses. Moreover, scores for perceived stress were significantly and positively related to eating attitude disorder and negatively associated with self-efficacy. Skewness and kurtosis of the distribution of the total scores of the dimensions of the study were calculated: skewness values were between −0.55 and 1.24; kurtosis values were between −1.17 and 1.10.

### 3.2. Path Analysis

**Figure 2:**
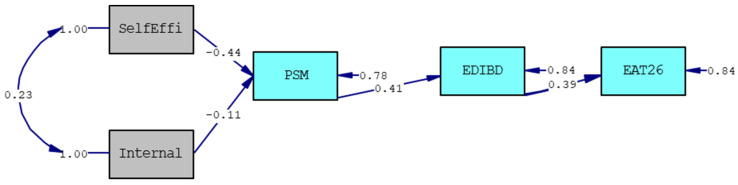
The Final Model (Model 1). Note. Selfeffi = self-efficacy; Internal = internal locus of control; PSM = perceived stress; EDIBD = body dissatisfaction; EAT26 = eating attitude disorder; chi-square = 9.05, df = 5, *p*-value = 0.10700, RMSEA = 0.052.

We tested our hypotheses using a path analysis. First, we tested the model on the whole sample (N = 300), obtaining the following fit indexes (Model 1: χ^2^_(5)_ = 9.23, *p* = 0.10; CFI = 0.98; RMSEA = 0.05; SRMR = 0.05) that suggest an excellent fit between model and data. The Model 1 is presented in Figure 2. The significant relationships are indicated by standardized β, which represents the intensity of the affection of the antecedents on the outcomes; as reported in Figure 2, both the antecedents—self-efficacy and internal locus of control—have a significant relationship on perceived stress that, in turn, affects body dissatisfaction, which is related to eating disorders.

Then, we compared the tested model in the three subsamples, aspiring fashion models (Model 2), athletes (Model 3) and control group (Model 4), obtaining the fit indexes reported in Table 4.

As shown in Table 4, even though the three models (2, 3 and 4) have very good fit indexes for all the three subsamples, comparing the fit indexes, Model 2 shows the best fit and the lower AIC; therefore, the hypothesized model could better explain the relationships between the variables in the group of models than in the other two groups. This confirmed our research hypotheses.

## 4. Discussion

To the best of our knowledge, the present study was the first to develop a path model of self-efficacy and internal locus of control variables as antecedents of perceived stress toward body dissatisfaction and eating attitude disorders, comparing fashion models to a group of athletes and a well-matched control group composed of females of the same age and cultural background. The results emphasized the importance of these dimensions in the context of body image. The research hypotheses were confirmed: in our model, we found substantial differences in the comparison of three subsamples. Although body dissatisfaction is a strong predictor of disordered eating, it is firstly important to underline that all participants in our sample did not concomitantly report severe levels of eating disorder symptomatology, as far as full syndrome anorexia or bulimia nervosa is concerned.

First of all, post hoc comparisons showed that the aspiring fashion models had significantly higher body dissatisfaction index, perceived stress, and risk of eating attitude disorder, and lower self-efficacy and internal locus of control than athletes and controls; moreover, they reported a BMI below 18, the cut-off for under-nutrition. Similarly, previous studies [41,42,43,55] reported that an elevated body image dissatisfaction and poor self-efficacy were related to health-related effects, such as stressor symptoms and greater risk of clinical eating disorders, in a sample of fashion models, compared to their peers. Social factors, such as the constant exposure to media images or the constant pressure to maintain a thin shape, can also contribute to the unhealthy lifestyle. The mean EAT-26 score was below the cut-off score of 20 that indicates risk of disordered eating attitudes but not the presence of clinically significant eating pathology. Generally, fashion models are chosen for this profession, because they have a constitution that lies at one lower level of the normal distribution of body types, to conform to the very restrictive shape and weight requests, without necessarily the risk of developing a nutritional disease. Furthermore, post hoc comparisons showed significantly lower scores in the group of controls on body dissatisfaction index and risk of eating attitude disorder than athletes. It seems that, although exposed to numerous risk factors, students care about their body and maintain correct eating habits [56]. Additionally, self-efficacy and internal locus of control were higher in the athletes, while perceived stress came out lower than controls. The present findings are consistent with research [22,23,24] that has shown the strong relationship between internal locus of control and self-efficacy, reducing, consequently, levels of stress. Tirmzai and Mughal [57], examining self-efficacy, sports participation and academic achievement among female athletes and female students, found higher self-efficacy in athletes. Based on the findings, it was concluded that sports participation increases self-efficacy and the confidence level of female athletes. Moreover, Nwankwo and colleagues [58], showed that high internal locus of control play significant role on psychological well-being of youth athletes, reporting lower indices of stress.

Pearson’s r correlation revealed very strong and positive relationships between body dissatisfaction, eating attitude disorder and perceived stress and negative relationship with self-efficacy. On the other hand, we found very strong relationships between the intrapersonal dimensions of self-efficacy and internal locus of control, which we assumed as the antecedents in our research hypotheses. Moreover, scores for perceived stress were significantly and positively related to eating attitude disorder and negatively associated with self-efficacy. According to the aforementioned literature, self-efficacy and internal locus of control, as intrapersonal dimensions to cope with environmental demands, represent some of the most important resource factors in stress appraisal processes. Because of these considerations, the impact of the stressful events has less negative effect on the health of the individual.

In the last investigation of the present study, comparing the tested path model in the three subsamples (aspiring fashion models, athletes and control group), we obtained very good fit indexes; more specifically, the model of aspiring fashion models showed the better fit index than in the other two groups. In many sports, according to Hausenblas and McNally [59], athletes are exposed to sociocultural pressure regarding body shape, and the maintenance of thinness is a crucial variable for performance. Furthermore, exercise intensity also contributes to low body fat. Chen and colleagues [60], observing the direct pathway in the relationship between body dissatisfaction and eating attitude disorder, showed that self-efficacy and depression symptoms partially mediated the relationship between these two dimensions in 654 adolescents.

In addition, as suggested in our results, aspiring fashion models can be much more exposed to the dangers of diet and exasperated thinness because of the few experiences, insecurity of future and probable competitive frustration that can diminish their ability to be resilient to stress. In a beauty contest, the competitive stress can cause candidates to intensify attempts to preserve their body weight below normal parameters. This attitude sometimes can bring conditions of food disorder. For this reason, the presence of skilled health workers in the field of nutrition and psychology can be extremely important in the field of fashion in relation to the training of girl models, where competitiveness and stress are unavoidable conditions.

## 5. Limitations

Notwithstanding the promising results, limitations of the study should be addressed. First, by using self-report inventories exclusively, data may be susceptible to selective or erroneous reporting. Second, the cross-sectional nature of the study does not permit us to state clearly the causal relationships between the variables.

Future research should establish whether the higher risk of eating disorders among aspiring fashion models observed in this study is an effect of the demand of the profession to maintain a thin figure or if the fashion model profession is specially chosen by girls already predisposed to eating disorders.

## 6. Conclusions

In conclusion, it is possible that health information on the psychological risks of eating disorders today has partially changed the attitudes of fashion models or, generally, of young females; however, they still remain at risk. For this reason, it is crucial to steer them away from unhealthy diet and potentially harmful weight-loss behaviors, providing the support of nutritionists and psychologists for the adoption of health eating and physical activity behaviors. It is also important to engage in these interventions, teachers, coaches and family members, as well as people working in the fashion industries.

## Figures and Tables

**Table 1:** Participants’ Anthropometric Characteristics. Mean ± SD (Range).

Groups	Age (Years)	BMI (kg/m^2^)	Weight (kg)	Height (cm)
Models	19.6 ± 2.01 (15–24)	18.2 ± 0.28 (17.8–19.1)	57.2 ± 3.95 (49–65.3)	176.4 ± 3.95 (169–183)
Athletes	19.8 ± 2.00 (17–24)	19.3 ± 0.38 (18.9–19.9)	61.5 ± 2.33 (56–66)	169.2 ± 5.12 (158–180)
Controls	19.4 ± 1.49 (17–22)	21.4 ± 1.02 (18.9–23.4)	61.6 ± 2.29 (55–66)	169.1 ± 4.07 (158–179)

**Table 2:** Analysis of Variance (ANOVA) Results.

Variable and Groups	M	SD	F	η*_p_*^2^
EDI–BD				
Models	29.46	6.21	131.99 *	0.47
Controls	14.70	3.67		
Athletes	22.51	8.47		
BITE				
Models	12.75	2.79		
Controls	12.93	3.66	0.80	0.05
Athletes	13.29	2.67		
PSM-9				
Models	33.75	10.26		
Controls	20.36	5.83	116.44 *	0.44
Athletes	19.80	4.62		
EAT-26				
Models	17.16	3.42		
Controls	11.89	2.10	73.19 *	0.33
Athletes	15.17	3.73		
SELF-EFFICACY				
Models	26.01	4.67		
Controls	34.01	9.87	84.68 *	0.36
Athletes	39.31	6.28		
FATALISM				
Models	9.73	1.60		
Controls	9.15	1.18	23.06 *	0.13
Athletes	8.42	1.27		
HETERO-DEPENDENCE				
Models	9.49	1.41		
Controls	8.88	1.91	35.98 *	0.19
Athletes	7.12	2.63		
INTERNALITY				
Models	6.66	0.80		
Controls	7.48	1.48	32.67 *	0.18
Athletes	8.24	1.70		

Notes: EDI–BD is Eating Disorder Inventory–Body Dissatisfaction subscale; BITE is Bulimic Investigatory Test; PSM-9 measures psychological stress; EAT-26 is Eating Attitudes Test. * *p* < 0.001.

**Table 3:** Correlation Coefficients (Pearson’s r).

Variable	1	2	3	4	5	6	7	8
1. EDI–BD	1							
2. BITE	−0.056	1						
3. PSM-9	0.406 *	0.173	1					
4. EAT-26	0.395 *	0.041	0.282 *	1				
5. Self-Efficacy	−0.239 *	−0.249 *	−0.460 *	−0.189 *	1			
6. Fatalism	−0.011	0.034	0.210 ^†^	0.125	−0.257 *	1		
7. Hetero-dependence	−0.041	0.012	0.182 ^†^	0.079	−0.231 *	0.219 *	1	
8. Internality	−0.050	−0.117	−0.209 *	−0.067	0.232 *	−0.208 *	−0.268 *	1

Notes: EDI–BD is Eating Disorder Inventory–Body Dissatisfaction subscale; BITE is Bulimic Investigatory Test; PSM-9 measures psychological stress; EAT-26 is Eating Attitudes Test. ^†^ *p* < 0.01; * *p* < 0.001.

**Table 4:** Comparison between Model 2, 3 and 4.

Models	χ^2^_(df)_	RMSEA (C.I.)	SRMR	CFI	AIC
Model 2 (models)	0.59_(5)_	0.0 (0.0–0.0)	0.02	1.00	20.59
Model 3 (athletes)	6.53_(5)_	0.05 (0.0–0.16)	0.06	0.93	26.33
Model 4 (controls)	4.57_(5)_	0.0 (0.0–0.13)	0.05	1.00	24.47

## Data Availability

The data that support the findings of this study are available from the corresponding author (D.D.C.), upon reasonable request.
